# ADSC-Exos containing MALAT1 promotes wound healing by targeting miR-124 through activating Wnt/β-catenin pathway

**DOI:** 10.1042/BSR20192549

**Published:** 2020-05-11

**Authors:** Lin He, Chan Zhu, Jing Jia, Xiao-Yan Hao, Xue-Yuan Yu, Xiang-Yu Liu, Mao-Guo Shu

**Affiliations:** 1 Department of Plastic, Aesthetic and Maxillofacial Surgery, The First Affiliated Hospital of Xi’an Jiaotong University, Xi’an 710061, P.R. China; 2 Department of Burns and Cutaneous Surgery, Xijing Hospital, The Fourth Military Medical University, Xi’an 710032, P.R. China

**Keywords:** adipose-derived stem cell, cutaneous wound healing, exosomes, MALAT1, miR-124

## Abstract

Cutaneous wound is a soft tissue injury that is difficult to heal during aging. It has been demonstrated that adipose-derived stem cells (ADSCs) and its secreted exosomes exert crucial functions in cutaneous wound healing. The present study aimed to elucidate the mechanism of exosomes derived from ADSCs (ADSC-Exos) containing MALAT1 in wound healing. ADSCs were isolated from human normal subcutaneous adipose tissues and identified by flow cytometry analysis. Exosomes were extracted from ADSC supernatants and MALAT1 expression was determined using qRT-PCR analysis. HaCaT and HDF cells were exposed to hydrogen peroxide (H_2_O_2_) for simulating the skin lesion model. Subsequently, CCK-8, flow cytometry, wound healing and transwell assays were employed to validate the role of ADSC-Exos containing MALAT1 in the skin lesion model. Besides, cells were transfected with sh-MALAT1 to verify the protective role of MALAT1 in wound healing. The binding relationship between MALAT1 and miR-124 were measured by dual-luciferase reporter assay. ADSC-Exos promoted cell proliferation, migration, and inhibited cell apoptosis of HaCaT and HDF cells impaired by H_2_O_2_. However, the depletion of MALAT1 in ADSC-Exos lose these protective effects on HaCaT and HDF cells. Moreover, miR-124 was identified to be a target of MALAT1. Furthermore, ADSC-Exos containing MALAT1 could mediate H_2_O_2_-induced wound healing by targeting miR-124 and activating Wnt/β-catenin pathway. ADSC-Exos containing MALAT1 play a positive role in cutaneous wound healing possibly via targeting miR-124 through activating the Wnt/β-catenin pathway, which may provide novel insights into the therapeutic target for cutaneous wound healing.

## Introduction

Cutaneous wound healing is a dynamic process and involves four precisely integrated and overlapping phases, including hemostasis, inflammation, proliferation and tissue remodeling [1,2]. The wound has a long healing cycle, the nonhealing cutaneous wounds without effective therapies can lead to severe clinical burden [3]. Ineffective skin wound healing is an important source of morbidity and mortality. Importantly, the risk of chronic skin wounds failing to heal increases as the age increases [4]. In the process of skin wound healing, the integration of skin cell differentiation, migration, proliferation and apoptosis plays a crucial role in skin tissue repair [5]. The wound after healing has the characteristic of regenerative epithelialization, which is related to two basic functions of keratinocytes, namely proliferation and migration [6]. Some growth factors, such as transforming growth factor β (TGF-β) and epidermal growth factor (EGF), can stimulate cell proliferation, differentiation and migration. Many signaling pathways play crucial role during cutaneous wound repair: the Wnt/β-catenin, Notch, Hedgehog and various growth factor/cytokine pathways [7]. The functions of these growth factors and signaling pathways in clinical research confirm their role in accelerating wound healing. Though certain progress is obtained in wound healing, the particular mechanism of cutaneous wound healing has not been fully clarified.

Mesenchymal stem cells (MSCs) are pluripotent stem cells with differentiation abilities, and have been widely used in the field of regenerative medicine because of their important role in enhancing the ability of various tissues to regenerate [8,9]. Numerous studies have reported the significant role of MSCs in neovascularization of ischemic tissue, including wound healing [10,11]. Exosomes are small multivesicular intraluminal vesicles ranging from 30 to 100 nanometers in diameter [12]. Exosomes are important paracrine mediators between MSCs and target cells, enabling cell-to-cell communication by delivering RNA and proteins to target cells [13,14]. Li et al. [15] reported that exosomes from adipose-derived stem cells (ADSCs) can potentially promote wound healing. Dalirfardouei et al. [16] suggested that the menstrual blood-derived MSCs-derived exosomes effectively ameliorated cutaneous nonhealing wounds. Ma et al.’s [17] findings also revealed that exosomes derived from ADSCs (ADSC-Exos) play a positive role in cutaneous wound healing possibly via Wnt/β-catenin signaling. MALAT1 may act as a transcriptional regulator for numerous genes, including some genes involved in cancer metastasis and cell migration, and it is involved in cell cycle regulation [18]. CD63 mediates signal transduction events that play a role in the regulation of cell development, activation, growth and motility [19]. CD9 functions in many cellular processes including differentiation, adhesion and signal transduction and expression of this gene plays a critical role in the suppression of cancer cell motility and metastasis [20]. However, the molecular mechanism of ADSCs in wound healing has not been fully investigated.

In the present study, we isolated exosomes from ADSCs and established a skin lesion model via exposure of HaCaT and HDF cells to hydrogen peroxide (H_2_O_2_), and then investigated the wound healing properties of exosomes derived from ADSCs. We demonstrated that ADSCs-Exos containing MALAT1 significantly attenuated H_2_O_2_-induced the suppression of cell proliferation, migration and the promotion of apoptosis via targeting miR-124 through Wnt/β-catenin pathway. Collectively, our data implied that MALAT1 may play critical regulatory roles in promoting cutaneous wound healing.

## Materials and methods

### Isolation and culture of ADSCs

Human normal subcutaneous adipose tissues (obtained in healthy people undergoing selective liposuction or undergoing surgical plastic surgery) were acquired from the First Affiliated Hospital of Xi’an Jiaotong University. The Research Ethics Committee of the First Affiliated Hospital of Xi’an Jiaotong University approved the study protocol, and informed consent was obtained from all the donors of adipose tissue. Then the adipose tissues were digested by collagenase type I for 60 min and was terminated using DME/F12 complete media. The cell-debris pellet was obtained by centrifugation at 1000 rpm for 5 min. The cells were cultured in a 5% CO_2_ humidified atmosphere at 37°C, and the medium was changed every 3 days. Upon reaching 80% confluence, the cells were defined as passage 1 and re-plated until the fourth passage for the following experiments.

### Flow cytometry analysis

The fourth-passage human ADSCs were tested by flow cytometry analysis. Briefly, the adherent cells were harvested by trypsinization, centrifuged at 800 rpm for 6 min, washed with sterile phosphate-buffered saline and resuspended. Cells were stained with specific fluorescein isothiocyanate–conjugated (FITC) monoclonal antibodies against CD29, CD44, CD34 and CD31 for 45 min at 4°C. For the negative control, an irrelevant antibody of the same isotype was used. Finally, the cells were analyzed using an FACS Calibur cytometer [21].

### Exosomes isolation and identification

Using exosome isolation reagent (RiboBio, Guangzhou, China), exosomes were extracted from ADSCs supernatants without cell-debris in accordance with the manufacturer’s guidelines. Subsequently, observation of isolated exosomes was monitored by means of transmission electron microscopy (TEM; JEOL Ltd., JEM2010-HT; Tokyo, Japan). The ADSCs-Exos were characterized by nanoparticle tracking analysis (NTA) and Western blotting. In the NTA assay, the size, distribution and the number of particles in the ADSCs-Exo were evaluated using a nanoparticle tracking analyzer (v3.1, Malvern Instruments, Ltd., Worcestershire, U.K.) [22]. Total RNA and protein were extracted using TRIzol-LS (Invitrogen, Carlsbad, CA, U.S.A.) and Exosomal Protein Extraction kit (Invitrogen), according to the manufacturer’s protocol, respectively. The ADSCs-Exo were analyzed by Western blotting using the following primary antibodies: anti-CD9 (1:1000, Abcam), anti-CD63 (1:1000, Abcam). Final exosomes were obtained and stored at −80°C for use for the following study.

### Cell culture and cell transfection

HaCaT cells were purchased from the American Type Culture Collection (ATCC, Manassas, U.S.A.) and HDF cells were obtained from ScienCell. Cells were cultured in Dulbecco’s modified Eagle’s medium (DMEM; HyClone, Logan) and 10% fetal bovine serum without exosomes (Gibco, Carlsbad, CA) and maintained in a humidified incubator at 37°C with 5% CO_2_.

For MALAT1 knockdown, MALAT1-targeting shRNAs (GenePharma, Shanghai, China) was synthesized to knockdown the expression of MALAT1 (sh-MALAT1). A negative control (GenePharma) was named as shNC. Anti-miR-124 was utilized to change the expression level of miR-124 in HaCaT and HDF cells. One control miRNAs (miR-NC) was used as negative controls for anti-miR-124. Cell transfection was then performed using Lipofectamine 2000 (Invitrogen) according to the manufacturer’s instructions.

### MTT assay

MTT assay was used to evaluate the survival of HaCaT and HDF cells with different H_2_O_2_ treatment (0, 100, 500, 1000, 1500 and 2000 µM). The details of MTT assay was performed as previously described [23].

### RNA isolation and real-time PCR

Total RNA was extracted with TRIzol Reagent (Invitrogen), followed by cDNA synthesis using a TransScript All-in-One First-Strand cDNA Synthesis SuperMix (Transgen Biotech, Beijing, China), was performed as described previously [24]. PCR was performed using a Bio-Rad PCR instrument (Bio-Rad, Hercules, CA, U.S.A.) with 2× Taq PCR Master Mix (Solarbio, Beijing, China) following the manufacturer’s instructions. The fold changes were calculated by means of relative quantification (2^−△△*C*_t_^ method). PCR primers are described as below:

MALAT1: forward 5′-GGGTGTTTACGTAGACCAGAACC-3′ and reverse 5′-CTTCCAAAAGCCTTCTGCCTTAG-3′; miR-124: forward 5′-TCGTTAAGGCACGCGGTG-3′ and reverse 5′-GTGCAGGGTCCGAGGT-3′; U6: forward 5′-CTCGCTTCGCAGCACA-3′ and reverse 5′-AACGCTTCACGAATTTGCGT-3′; GAPDH forward 5′-GCACCGTCAAGGCTGAGAAC-3′ and reverse 5′-ATGGTGGTGAAGACGCCAGT-3′.

### CCK-8 assay

The capacity of cell proliferation was assessed using CCK-8 (Beyotime Biotechnology). Cells were planted into 96-well plates (5000 cells/well) containing culture medium. Subsequently, cells were exposed to different concentrations of H_2_O_2_ at indicated times. CCK-8 assay was carried out by adding 10 µl of CCK-8 reagent into each well. After incubated in an incubator for another 2 h, cell proliferation was measured by testing the absorbance at 450 nm using a microplate reader (Bio-Rad Laboratories, Inc., Hercules, CA, U.S.A.).

### Migration assay

The migratory properties of HaCaT and HDF cells were tested by two methods. (1) For the scratch wound healing assay, HaCaT and HDF cells were seeded into plastic six-well plates at the density of 5 × 10^5^ cells per well and cultured for 12 h. Cells were treated with 500 µM of H_2_O_2_ for 4 h when they reached a confluence of 80%. After discarding the culture medium, uniform scratch wounds were scraped by a sterile pipette tip. Each well was washed with PBS, and then supplemented with basal DMEM containing H_2_O_2_, H_2_O_2_+ADSC-Exo, H_2_O_2_+ADSC-Exo-shNC, H_2_O_2_+ADSC-Exo-shMALAT1, H_2_O_2_+ADSC-Exo-shMALAT1+anti-miR-124, respectively. Images for each scratch were observed by microscope and captured at 0 and 24 h after scratching. (2) For the transwell assay, transwell chambers (8 µM, Corning Incorporated, Corning, NY) without Matrigel were applied following the manufacturer’s protocol. Cells were treated with H_2_O_2_, H_2_O_2_+ADSC-Exo, H_2_O_2_+ADSC-Exo-shNC, H_2_O_2_+ADSC-Exo-shMALAT1, H_2_O_2_+ADSC-Exo-shMALAT1+anti-miR-124, respectively. Subsequently, the harvested cells were suspended in 200 µl of serum-free media and were added into the upper chamber, and the lower chamber was filled with normal growth media. After 24 h incubation, the migrated cells were fixed with 4% formaldehyde and stained with 0.1% Crystal Violet solution [17].

### Dual-luciferase reporter assay

The 3′UTR of MALAT1 gene containing the predicated binding sites for miR-124 were amplified using PCR. The fragment was inserted into the multiple cloning sites in the pMIR-REPORT luciferase miRNA expression reporter vector (Ambion, Austin, U.S.A.). Then, HEK293T cells were co-transfected with 0.1 μg of luciferase reporter vectors comprising wild-type or mutant type of 3′UTR of MALAT1 and either miR-124 mimic or miR-control by Lipofectamine 2000 (Invitrogen, Carlsbad, U.S.A.). Relative luciferase activity was calculated by normalizing the firefly luminescence to the *Renilla* luminescence using the Dual-Luciferase Reporter Assay System (Promega, Madison, WI, U.S.A.) according to the manufacturer’s instructions at 48 h post-transfection.

### Statistical analysis

Experiments were independently performed at least three times. All data were presented as mean ± standard deviation (SD) and the differences among multiple groups were analyzed by Student’s *t* test or one-way analysis of variance (ANOVA). The differences were considered to be statistically significant as *P-*value <0.05.

## Results

### Isolation and characterization of ADSCs and ADSC-Exos

**Figure 1:**
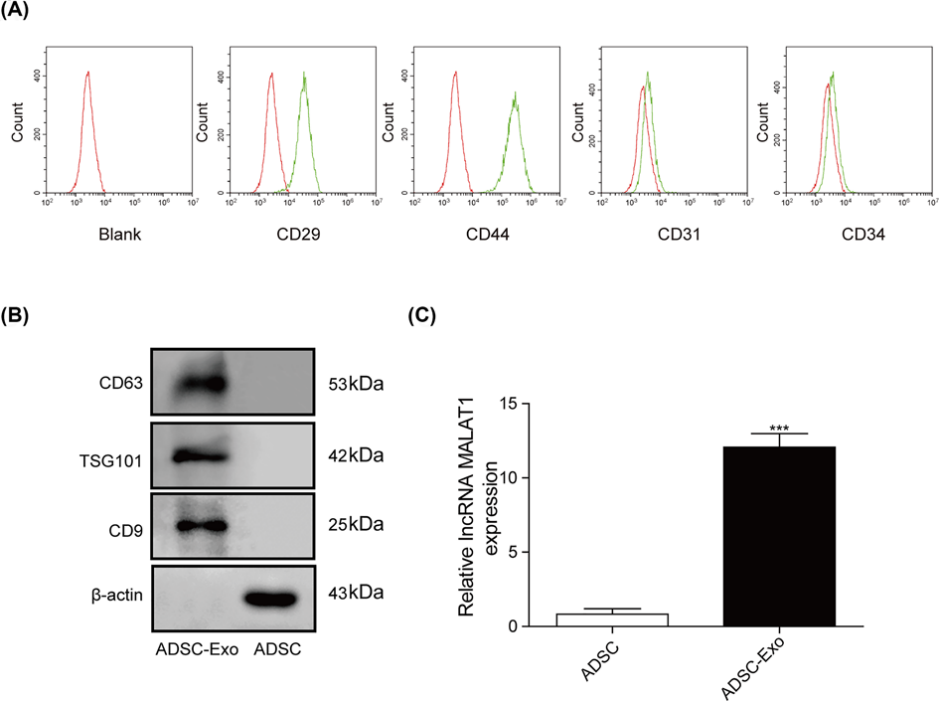
Isolation and characterization of ADSCs and ADSC-Exos (**A**) The expression of biomarkers (CD34, CD31, CD29 and CD44) were detected by FACS. (**B**) The exosomes’ marker proteins (CD63, TSG101 and CD9) detected by Western blot. (**C**) The expression level of MALAT1 was measured using qRT-PCR assay. ***P<0.001.

After primary isolation, ADSCs were cultured for 24 h. Subsequently, we observed that most of the adherent cells were in spindle-like shape during cell culture using a microscope (×40 magnification) (Supplementary Figure S1). Besides, flow cytometry analysis was performed to measure the relevant biomarkers. As shown in Figure 1A, CD29 and CD44 (MSC markers) were highly expressed in ADSCs at passage 4, while the expression level of CD34 and CD31 (hematopoietic cell markers) were not expressed in the ADSCs (Figure 1B). In addition, exosomes purified from ADSCs culture supernatants were characterized by TEM, and the results showed that exosomes were round membrane-bound vesicles with 30–100 nm diameter (Supplementary Figure S2). Meanwhile, CD9, TSG101 and CD63, which seemed to be the exosomes marker protein, were also detected in the exosomes as expected (Figure 1B). Furthermore, the gene level of MALAT1 was highly expressed in ADSC-exos compared with ADSCs (Figure 1C).

### Construction of the skin lesion model

**Figure 2:**
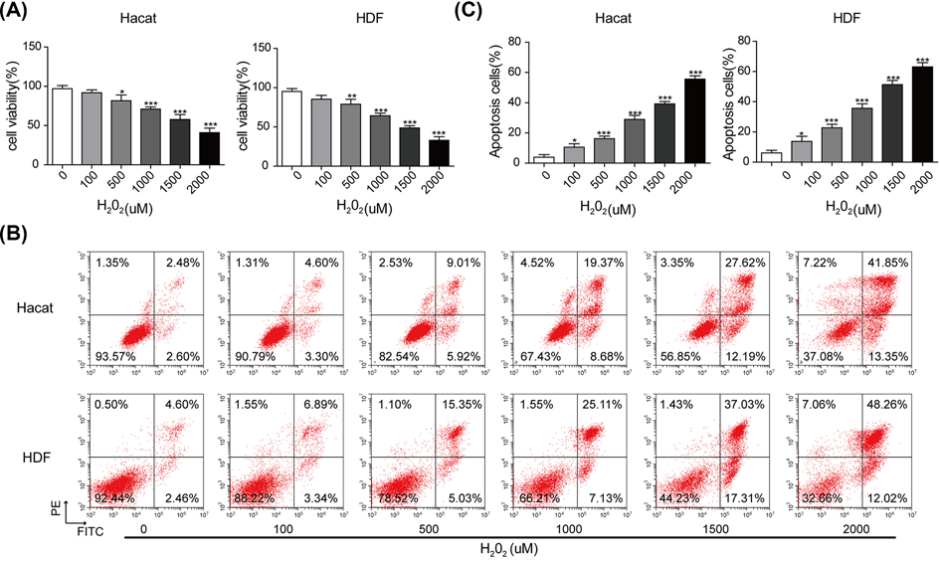
Construction of the skin lesion model *in vitro* (**A**) Cell viability assessed by MTT assay. (**B**) Apoptotic rate of HaCaT and HDF cells exposed with different concentrations of H_2_O_2_ examined by flow cytometry assay. (**C**) The percentage of apoptosis cell for HaCaT and HDF cells. *P<0.05, **P<0.01, ***P<0.001.

To simulate the skin lesion model *in vitro*, HaCaT and HDF cells were treated with H_2_O_2_ at different concentrations of 0, 100, 500, 1000, 1500 and 2000 µM. As shown in Figure 2A, cell viability of HaCaT and HDF cells was significantly inhibited by the application of H_2_O_2_ in a dose-dependent manner, indicating that H_2_O_2_ could induce skin lesions. Consistently, the results obtained from flow cytometry assay (Figure 2B,C) showed that H_2_O_2_ also promoted the apoptosis of HaCaT and HDF cells, compared with that of control. Besides, as shown in Western blot analysis (Supplementary Figure S3), the results revealed that the increased expression of Caspase-3/Bax and the decreased expression of Bcl-2 were detected, with the enhanced concentrations of H_2_O_2_.

### ADSC-Exos attenuate the suppression of cell proliferation, migration and the promotion of cell apoptosis in HaCaT and HDF cells impaired by H_2_O_2_

**Figure 3:**
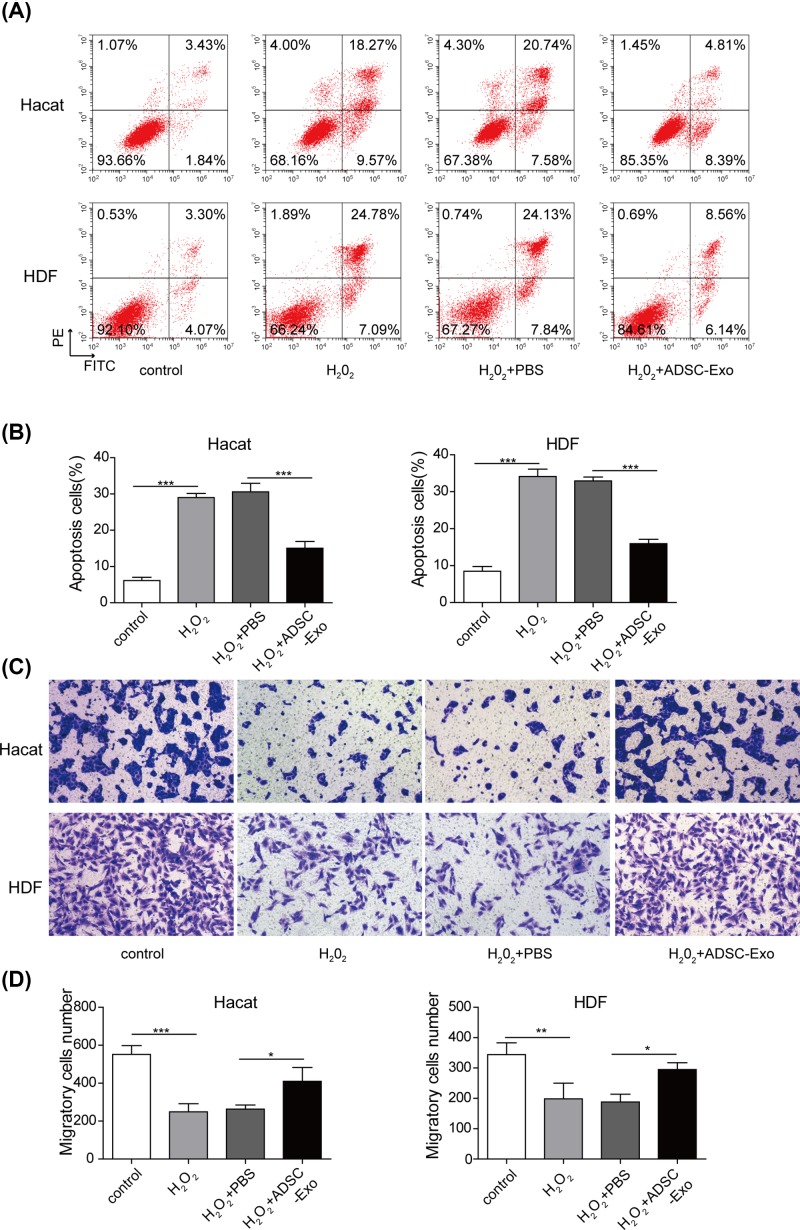
Effects of ADSC-Exos on cell proliferation, apoptosis and migration of HaCaT cells impaired by H_2_O_2_ (**A**) Apoptosis of HaCaT and HDF cells was evaluated by flow cytometry assay. (**B**) The percentage of apoptosis cell for HaCaT and HDF cells. (**C,D**) The capacity of cell migration in HaCaT and HDF cells was analyzed by transwell assay. *P<0.05, **P<0.01, ***P<0.001.

To explore the biological functions of ADSC-Exos in the cutaneous wound healing, the following experiments were employed. As shown in Supplementary Figure S4, cell proliferation was obviously repressed by H_2_O_2_, while the suppressive effects were reversed after the addition of ADSC-Exos. Meanwhile, flow cytometry also presented that ADSC-Exos significantly reduced the apoptotic rate of HaCaT and HDF cells induced by H_2_O_2_ (Figure 3A,B). Moreover, the capacity of cell migration was also determined using the scratch wound healing (Supplementary Figure S5) and transwell assays (Figure 3C,D). As expected, the migratory ability of HaCaT and HDF cells were impaired by H_2_O_2_, but the migratory ability was increased after adding the ADSC-Exos. These findings implied the protective role of ADSC-Exos in the cutaneous wound healing.

### ADSC-Exos knocking down MALAT1 lose the protective effects on HaCaT and HDF cells impaired by H_2_O_2_

**Figure 4:**
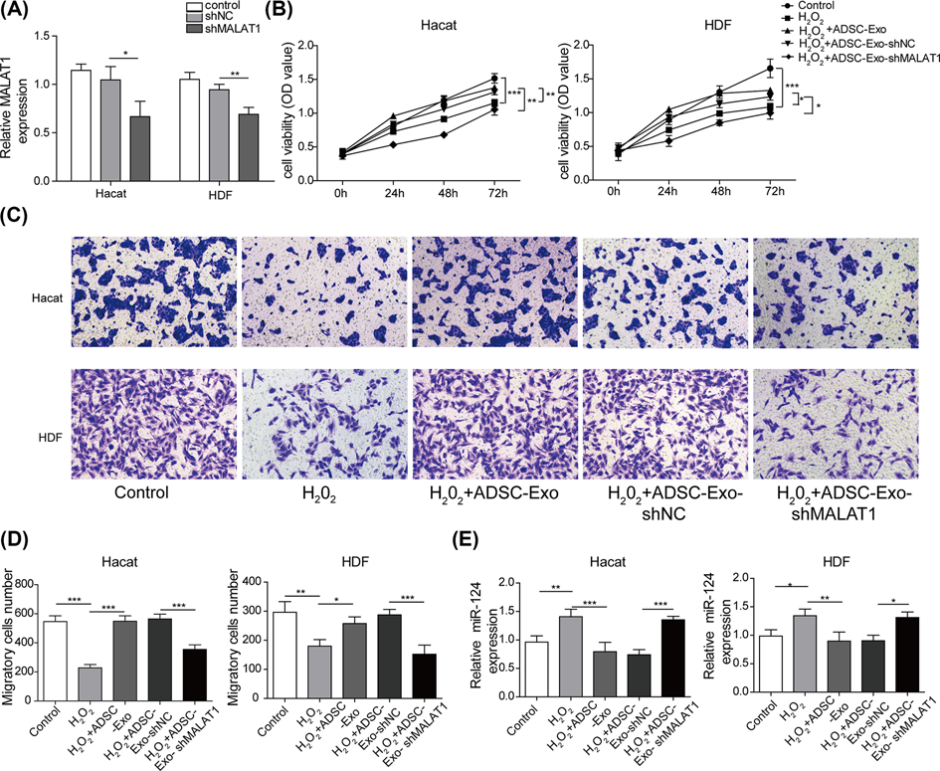
ADSC-Exos knocking down MALAT1 lose protective effects on HaCaT and HDF cells (**A**) The expression level of MALAT1 was measured by qRT-PCR assay. (**B**) Proliferation of HaCaT and HDF cells treated with H_2_O_2_, H_2_O_2_+ADSC-Exo, H_2_O_2_+ADSC-Exo-shNC or H_2_O_2_+ADSC-Exo-shMALAT1 were evaluated by CCK-8 assay. (**C,D**) Migration of HaCaT and HDF cells treated with H_2_O_2_, H_2_O_2_+ADSC-Exo, H_2_O_2_+ADSC-Exo-shNC or H_2_O_2_+ADSC-Exo-shMALAT1 were analyzed by Transwell assay. (**E**) The expression level of miR-124 was performed using qRT-PCR analysis. *P<0.05, **P<0.01, ***P<0.001.

To further validate whether ADSC-Exo containing MALAT1 play a role in mediating skin lesion model, we performed a pretreatment of ADSCs with MALAT1 knockdown, followed by collection of conditioned supernatants for exosomal extraction, namely ADSC-Exo-shMALAT1. As shown in Figure 4A, qRT-PCR assay confirmed that the expression level of MALAT1 was down-regulated after MALAT1 knockdown (for HaCaT approximately 40%, for HDF approximately 30%). As shown in Figure 4B, ADSC-Exos dramatically attenuated the impairment of H_2_O_2_ treatment, on cell proliferation in HaCaT and HDF cells. However, the positive effect of ADSC-Exos was significantly inhibited after knocking down MALAT1 (Figure 4B). Similarly, flow cytometry assay also showed dramatically decreased apoptosis of HaCaT and HDF cells after adding ADSC-Exos and obvious increased apoptosis after MALAT1 knockdown (Supplementary Figure S6). Additionally, the results of scratch wound healing assay also implied that the migratory ability of HaCaT and HDF cells were decreased by H_2_O_2_, but were increased after adding the ADSC-Exos. However, the promotion of cell migration induced by ADSC-Exos was reversed within MALAT1 knockdown (Supplementary Figure S7). Similar results were obtained from transwell assay (Figure 4C,D). Furthermore, the expression level of miR-124 detected by qRT-PCR was decreased after adding ADSC-Exos, but was increased after MALAT1 knockdown (Figure 4E). Taken together, ADSCs-Exo containing MALAT1 exert a protective role in the cutaneous wound healing.

### MALAT1 acts as a sponge of miR-124

**Figure 5:**
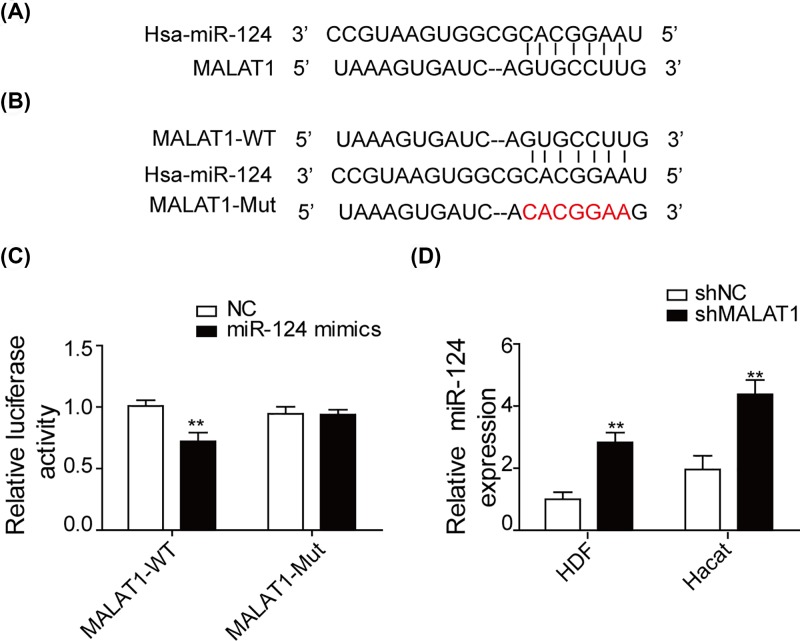
MALAT1 acts as a sponge of miR-124 (**A**) Analysis of binding sites of MALAT1 and miR-124 using bioinformatics software. (**B**) The wild-type and mutant-type of the complementary binding sequences between MALAT1 and miR-124. (**C**) The binding relationship between MALAT1 and miR-124 was employed using dual-luciferase reporter assay. (**D**) qRT-PCR was used to detect the expression of miR-124 after transfection with sh-MALAT1. **P<0.01.

We used bioinformatics software to predict the binding sites of MALAT1 and miR-124, which was shown in Figure 5A. The complementary nucleotides between miR-124 and the seed region in MALAT1 reporter plasmids (WT) of ‘GUGCCUUG’ were replaced by ‘CACGGAAU’ to construct MALAT1 mutant (Figure 5B). To identify whether MALAT1 directly targets miR-124, luciferase activity was assessed after co-transfection. As shown in Figure 5C, miR-124 mimic markedly decreased the luciferase activity of HEK-293T transfected with plasmid containing MALAT1-WT but not MALAT1-Mut. We also utilized qRT-PCR to study the expression level of miR-124 in shNC and shMALAT1 groups, which was shown in Figure 5D. There was higher expression level of miR-124 in the shMALAT1 group compared with shNC group.

### ADSC-Exos containing MALAT1 mediates H_2_O_2_-induced wound healing by targeting miR-124 through activating the Wnt/β-catenin pathway

**Figure 6:**
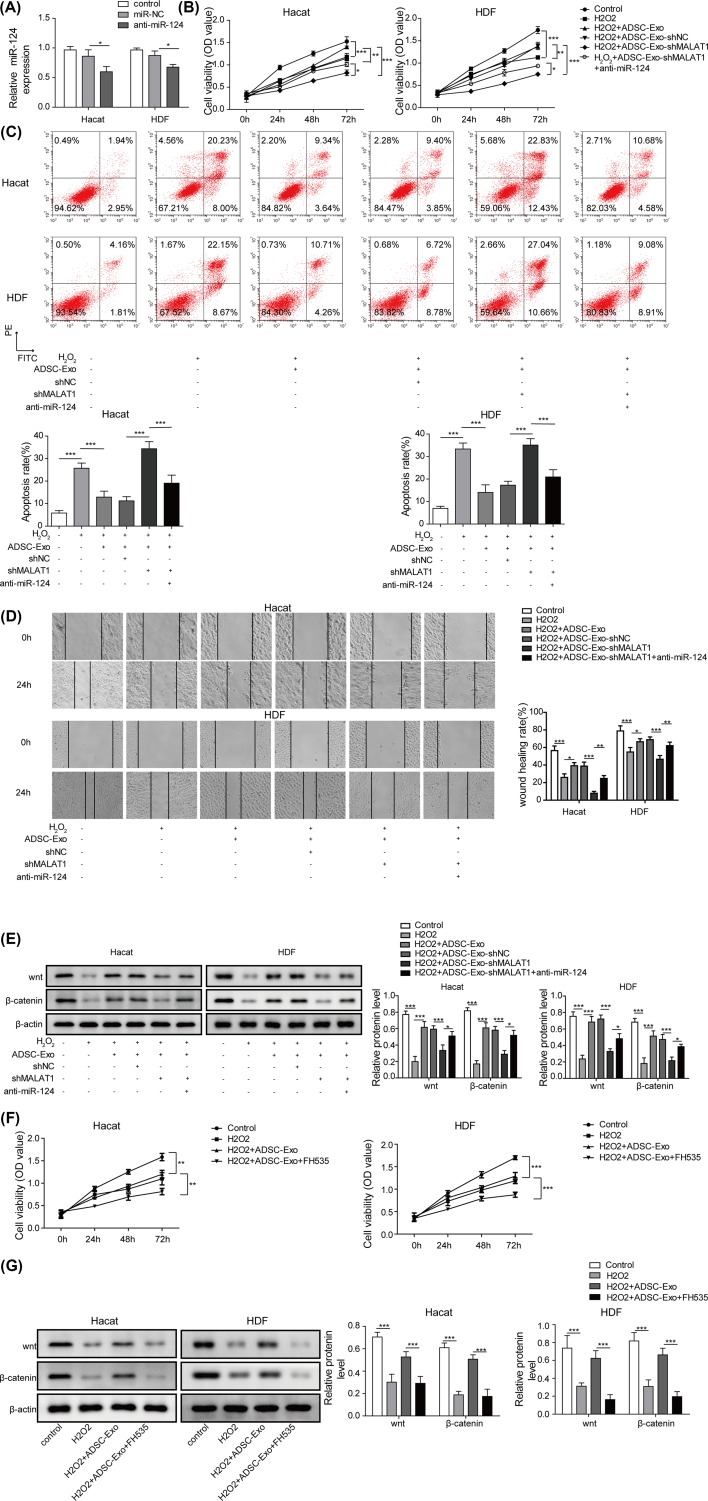
ADSC-Exos containing MALAT1 mediates H_2_O_2_ induced wound healing by targeting miR-124 through activating Wnt/β-catenin pathway (**A**) The expression level of miR-124 was detected by qRT-PCR after transfected with anti-miR-124. (**B**) CCK-8 assay was performed to assess the cell proliferation of HaCaT and HDF cells treated with H_2_O_2_, H_2_O_2_+ADSC-Exo, H_2_O_2_+ADSC-Exo-shNC, H_2_O_2_+ADSC-Exo-shMALAT1, H_2_O_2_+ADSC-Exo-shMALAT1+anti-miR-124. (**C**) Flow cytometry was subjected to evaluate cell apoptosis of HaCaT and HDF cells treated with H_2_O_2_, H_2_O_2_+ADSC-Exo, H_2_O_2_+ADSC-Exo-shNC, H_2_O_2_+ADSC-Exo-shMALAT1, H_2_O_2_+ADSC-Exo-shMALAT1+anti-miR-124. (**D**) Migration of HaCaT and HDF cells treated with H_2_O_2_, H_2_O_2_+ADSC-Exo, H_2_O_2_+ADSC-Exo-shNC, H_2_O_2_+ADSC-Exo-shMALAT1, H_2_O_2_+ADSC-Exo-shMALAT1+anti-miR-124 were analyzed by the scratch wound healing assay. (**E**) The expression of Wnt/β-catenin signals were determined by western blot. (**F**) Cell proliferation of HaCaT and HDF cells treated with FH535 were evaluated by CCK-8 assay. (**G**) Wnt/β-catenin signal pathway of HaCaT and HDF cells treated with H_2_O_2_, H_2_O_2_+ADSC-Exo or H_2_O_2_+ADSC-Exo+FH535 were monitored by western blot. *P<0.05, **P<0.01, ***P<0.001.

It has been demonstrated that Wnt/β-catenin signaling is related with wound healing [25], following that, whether Wnt/β-catenin signaling is also involved in ADSC-Exo containing MALAT1 in wound healing was further explored. As shown in Figure 6A, the expression level of miR-124 was markedly decreased in anti-miR-124 group compared with that of control. Subsequently, the results of CCK-8 assay presented that ADSC-Exos relieved the inhibition of H_2_O_2_ on proliferation ability of HaCaT and HDF cells, while MALAT1 knockdown recovered the impairment of H_2_O_2_ on cell viability. However, the capacity of cell proliferation was recovered after silencing of miR-124 (Figure 6B). Similarly, ADSC-Exos relieved the apoptosis induced by H_2_O_2_ in HaCaT and HDF cells, but the effects were blocked by MALAT1 knockdown. Moreover, the apoptosis was decreased after silencing the expression of miR-124 (Figure 6C). Besides, the inhibitory effect of H_2_O_2_ on migration ability of HaCaT and HDF cells was restrained after adding ADSC-Exos, while down-regulation of MALAT1 impaired the protection of ADSC-Exos on the migration ability. However, the effects were recovered by silencing of miR-124 (Figure 6D). In addition, Wnt/β-catenin signaling was detected using Western blot and the results showed that Wnt/β-catenin signaling was activated after adding ADSC-Exos, and MALAT1 knockdown further caused the inactivation of Wnt/β-catenin signaling pathway. However, silencing of miR-124 regained the promotion of Wnt/β-catenin signaling (Figure 6E). Furthermore, FH535, an inhibitor of Wnt/β-catenin signaling, notably impaired the protection of ADSC-Exos on cell proliferation and migration (Figure 6F and Supplementary Figure S8). As expected, compared with control group, FH535 significantly blocked the activation of Wnt/β-catenin signaling pathway (Figure 6G).

## Discussion

Although medical technology has made great progress in promoting wound healing over the past decades, scar formation during healing remains a challenging clinical problem [26]. Cell therapy has shown potential to enhance skin wound healing and decrease fibrosis after wounding [27]. The potential use of MSCs has been a hot topic in tissue repair studies [28]. ADSCs have the characteristics of pluripotent differentiation and extended proliferation ability, and can be harvested in large quantities [29]. Previous studies have indicated that ADSCs secrete several trophic factors and have a degree of immune privilege because of their ability to suppress T-cell-mediated responses that cause tissue rejection [30]. ADSCs have been successfully used in the treatment of soft tissue defects, scars and burns by accelerating and improving the quality of the wound healing process. ADSCs promote angiogenesis, epithelial cell migration, growth factor secretion and differentiation into multiple lineages [31], thus enhancing wound healing and reducing scar formation. However, the exact mechanism still remains elusive. In the present study, we exposed HaCaT and HDF cells to H_2_O_2_ to simulate a skin lesion model. The results of increased apoptosis, increased Bax expression and decreased Bcl-2 expression confirmed the validity of the model.

Exosomes are released from cells and participate in many biological and pathological processes. Since exosomes can carry messenger RNA, microRNAs and proteins, which can be transferred to target cells, the novel role of exosomes as cell communication bodies has been verified in many researches. The promotive role of exosomes in the proliferation, migration and angiogenesis process has been demonstrated. There is increasing evidence showing that the ability of ADSCs to stimulate skin fibroblast proliferation during wound healing might be due to the presence of growth factors secreted by ADSCs, especially exosomes. Therefore, we aimed to investigate whether ADSC-Exos exert protective functions in wound healing. In the present study, the existence of exosomes derived from ADSC was evidenced by CD63, TSG101 and CD9 expression. Interestingly, we observed that exosomes derived from ADSCs significantly promoted proliferation, migration and inhibited apoptosis of HaCaT and HDF cells induced by H_2_O_2_, these findings are consistent with data reported by other researchers, who have indicated that human ADSCs accelerate the repair process in various adult tissues and improve the quality of repair via exosomes [32–34]. Further investigation revealed that the positive effects of ADSC-Exos were blocked by MALAT1 knockdown. To explore the molecular mechanism of ADSC-Exos in wound healing, the following researches were conducted.

MALAT1 was one of the first identified lncRNAs associated with human diseases, which was originally described to be associated with metastasis of lung cancer [35]. It has been reported that MALAT1 drives tumorigenesis through the promotion of tumor cell proliferation. Moreover, overexpression of MALAT1 results in increased cell migration *in vitro* [36]. Depletion of MALAT1, on the other hand, inhibits cell motility *in vitro* and significantly limits metastasis formation in mouse cancer models [37]. Besides, it has also been reported that MALAT1 is involved in diabetes-induced microvascular dysfunction and regulates retinal endothelial cell proliferation, migration and tube formation [38]. Previous study reported that MALAT1 increased cell migration via modulating miR-140 expression in a uveal melanoma cell line [39]. MALAT1 can also up-regulate the expression of miR-22-3p target genes CXCR2 and Akt [40]. However, the exact role of MALAT1 on wound healing has not been clearly identified despite its extensive investigation in the cancer. Herein, we investigated the potential role of lncRNA MALAT1 in wound healing via MALAT1 knockdown in ADSC-Exos. The expression level of miR-124 was significantly increased after MALAT1 knockdown. Dual-luciferase reporter assay also verified the direct binding between MALAT1 and miR-124.

As previously described, Zhang et al. [41] found that miR-124 inhibited keratinocyte proliferation, collagen biosynthesis and activation of Wnt/β-catenin by targeting SERP1. Yang et al. [42] reported that miR-124 inhibited proliferation, migration and invasion of malignant melanoma (A375) cells. The function of Wnt/β-catenin signaling pathway has been widely studied; it is involved not only in cell proliferation and cell cycle, but also in wound healing [43,44]. In addition, the Wnt/β-catenin pathway has been shown to regulate keratinocyte proliferation and apoptosis in psoriasis lesions. Therefore, we investigated the involvement of Wnt/β-catenin pathway in wound healing. In our results, MALAT1 knockdown caused the inactivation of Wnt/β-catenin signaling pathway. However, silencing of miR-124 recovered the Wnt/β-catenin signaling pathway. ADSC-Exos induced cell viability was inhibited after adding FH535, an inhibitor of Wnt/β-catenin signaling pathway. Similarly, the migratory ability of HaCaT and HDF cells was also repressed by FH535 treatment. These results suggested that Wnt/β-catenin pathway was involved in the wound healing, which was activated by MALAT1.

Finally, we have shown that ADSC-Exos containing MALAT1 significantly promote cell proliferation, migration and inhibit cell apoptosis in the skin lesion models. miR-124 is a target gene of MALAT1 and its downexpression could attenuate the protective effects of ADSC-Exos on cutaneous cells. Moreover, MALAT1 significantly activates the Wnt/β-catenin pathway. These findings implied that ADSC-Exos containing MALAT1 mediates H_2_O_2_-induced wound healing by targeting miR-124 and activating the Wnt/β-catenin pathway. Nevertheless, the effect and underling mechanism of MALAT1 and miR-124 on the development of wound healing are still in their infancy. Further research is needed to better understand the mechanism whereby MALAT1 regulates the progression of wound healing before MALAT1-based therapeutics can be taken into clinical practice.

As a pilot study, we are currently screening the mechanism of ADSC-Exos containing MALAT1 in wound healing by using ADSCs and its secreted exosomes only at the cellular level. Our future *in vivo* molecular intervention experiments will extend our current findings.

## Supplementary Material

Supplementary Figures S1-S8

## Abbreviations

ADSC: adipose-derived stem cell
ADSC-Exos: exosomes derived from ADSC
CCK-8: cell counting kit-8
DMEM: Dulbecco’s modified Eagle’s medium
GAPDH: glyceraldehyde-3-phosphate dehydrogenase
H_2_O_2_: hydrogen peroxide
MALAT1: metastasis associated lung adenocarcinoma transcript 1
MSC: mesenchymal stem cell
NTA: nanoparticle tracking analysis
qRT-PCR: quantitative real-time polymerase chain reaction
SERP1: stress-associated endoplasmic reticulum protein 1
