# Supplementary material for: ADSC-Exos containing MALAT1 promotes wound healing by targeting miR-124 through activating Wnt/β-catenin pathway
Source: Biosci Rep. 2020 May 11;40(5):BSR20192549. doi: 10.1042/BSR20192549 (PMC7214401; doi:10.1042/BSR20192549)
Supplement: Supplementary Figures S1-S8 [file BSR-2019-2549_supp.pdf]

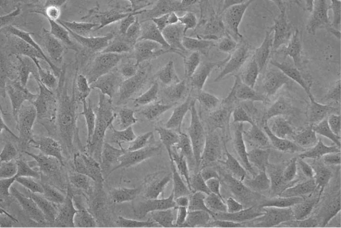

ADSC

Supplementary Figure S1 Spindle ADSCs were observed through the microscope. ×40 magnification.

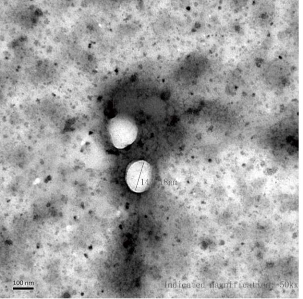

ADSC-Exo

Supplementary Figure S2 Transmition electron microscopy photomicrographs of exosomes. Scale bar, 100 nm.

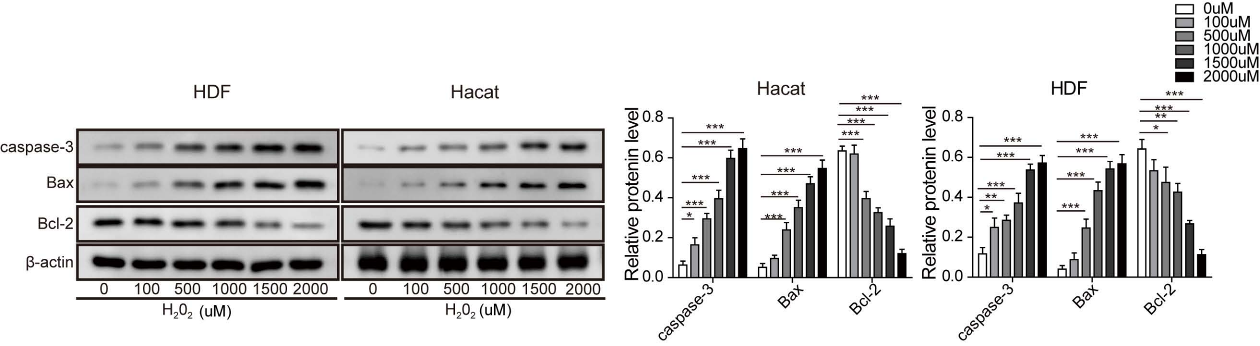

HDF
Hacat
caspase-3
Bax
Bcl-2
β-actin
0 100 500 1000 1500 2000
H2O2 (uM)
Hacat
HDF
Relative protenin level
0.0
0.2
0.4
0.6
0.8
caspase-3
Bax
Bcl-2
*
**
***
0uM
100uM
500uM
1000uM
1500uM
2000uM

Supplementary Figure S3 The Caspase3, Bax and Bcl-2 expression were detected by western blot assay.

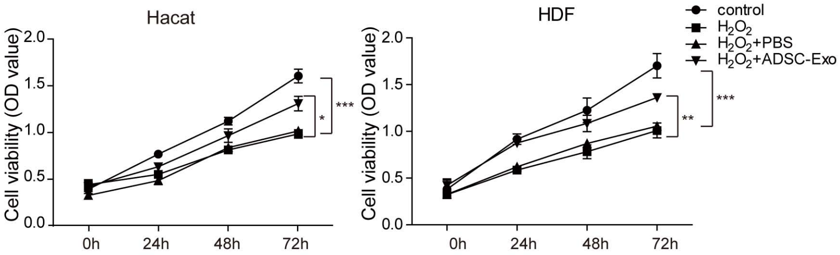
Hacat
HDF
Cell viability (OD value)
2.0
1.5
1.0
0.5
0.0
0h
24h
48h
72h
*
***
**
control
H_2O_2
H_2O_2+PBS
H_2O_2+ADSC-Exo

Supplementary Figure S4 Cell proliferation of HaCaT and HDF cells was evaluated by CCK-8 assay.

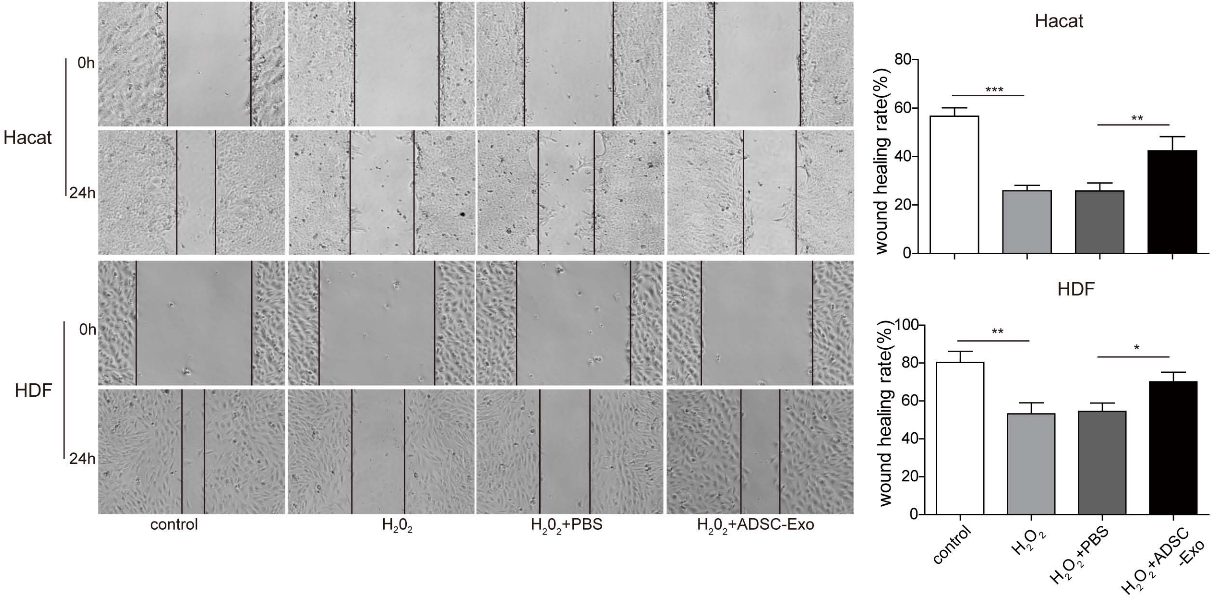

Hacat
0h
24h
HDF
0h
24h
control
H202
H202+PBS
H202+ADSC-Exo
Hacat
wound healing rate(%)
80
60
40
20
0
***
**
HDF
wound healing rate(%)
100
80
60
40
20
0
**
*
control
H2O2
H2O2+PBS
H2O2+ADSC-Exo

Supplementary Figure S5 The capacity of cell migration in HaCaT and HDF cells was analyzed by the scratch wound healing assay.

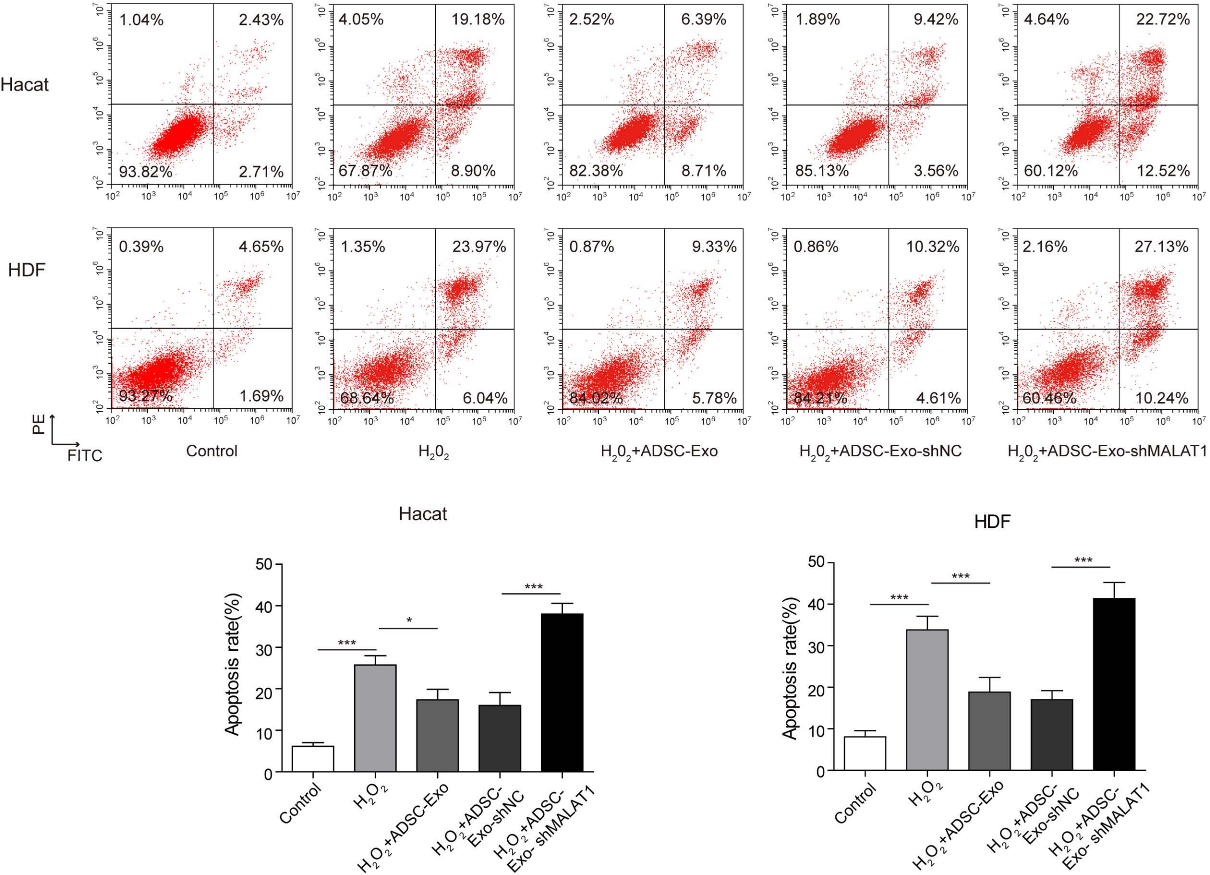

Hacat
HDF
PE
FITC
Control
H2O2
H2O2+ADSC-Exo
H2O2+ADSC-Exo-shNC
H2O2+ADSC-Exo-shMALAT1
1.04%
2.43%
93.82%
2.71%
4.05%
19.18%
67.87%
8.90%
2.52%
6.39%
82.38%
8.71%
1.89%
9.42%
85.13%
3.56%
4.64%
22.72%
60.12%
12.52%
0.39%
4.65%
93.27%
1.69%
1.35%
23.97%
68.64%
6.04%
0.87%
9.33%
84.02%
5.78%
0.86%
10.32%
84.21%
4.61%
2.16%
27.13%
60.46%
10.24%
Apoptosis rate(%)
H2O2+ADSC-Exo-shNC
H2O2+ADSC-Exo- shMALAT1
***
*

Supplementary Figure S6 Apoptosis of HaCaT and HDF cells treated with H_2O_2, H_2O_2+ADSC-Exo, H_2O_2+ADSC-Exo-shNC or H_2O_2+ADSC-Exo-shMALAT1 were monitored by flow cytometry assay.

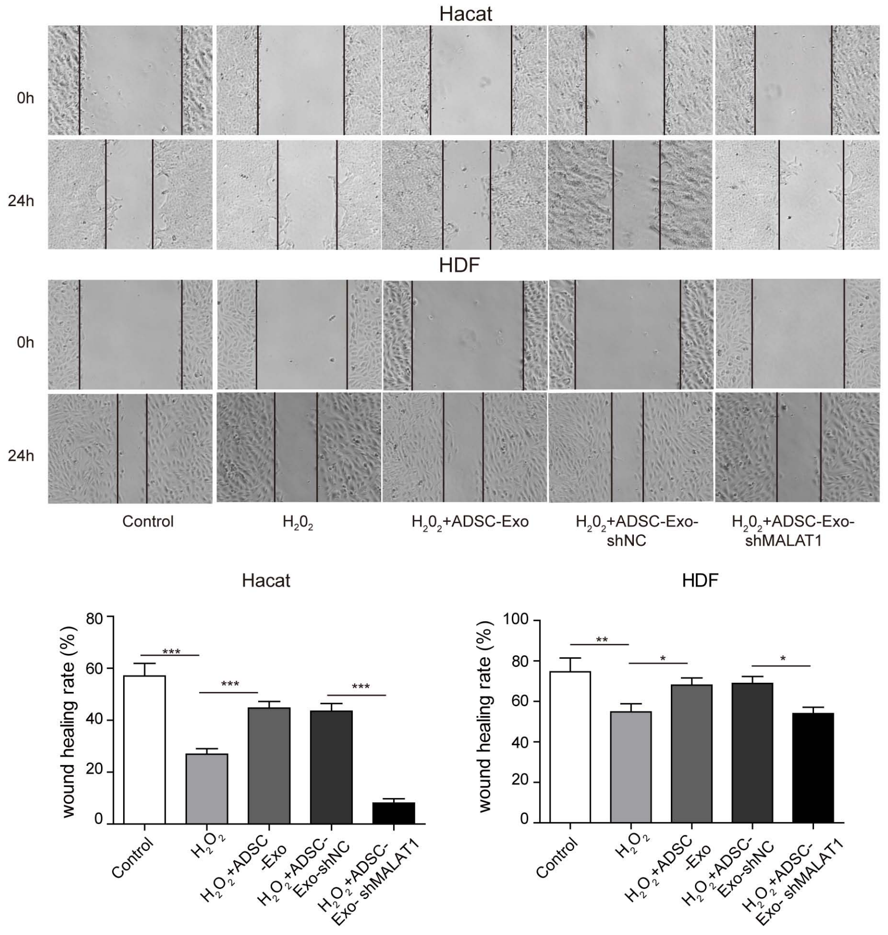
Hacat
0h
24h
HDF
0h
24h
Control
H_20_2
H_20_2+ADSC-Exo
H_20_2+ADSC-Exo-shNC
H_20_2+ADSC-Exo-shMALAT1
Hacat
wound healing rate (%)
0
20
40
60
80
***
***
***
Control
H_2O_2
H_2O_2+ADSC-Exo
H_2O_2+ADSC-Exo-shNC
H_2O_2+ADSC-Exo- shMALAT1
HDF
wound healing rate (%)
0
20
40
60
80
100
**
*
*
Control
H_2O_2
H_2O_2+ADSC-Exo
H_2O_2+ADSC-Exo-shNC
H_2O_2+ADSC-Exo- shMALAT1

Supplementary Figure S7 Migration of HaCaT and HDF cells treated with H_2O_2, H_2O_2+ADSC-Exo, H_2O_2+ADSC-Exo-shNC or H_2O_2+ADSC-Exo-shMALAT1 were analyzed by the scratch wound healing assay.

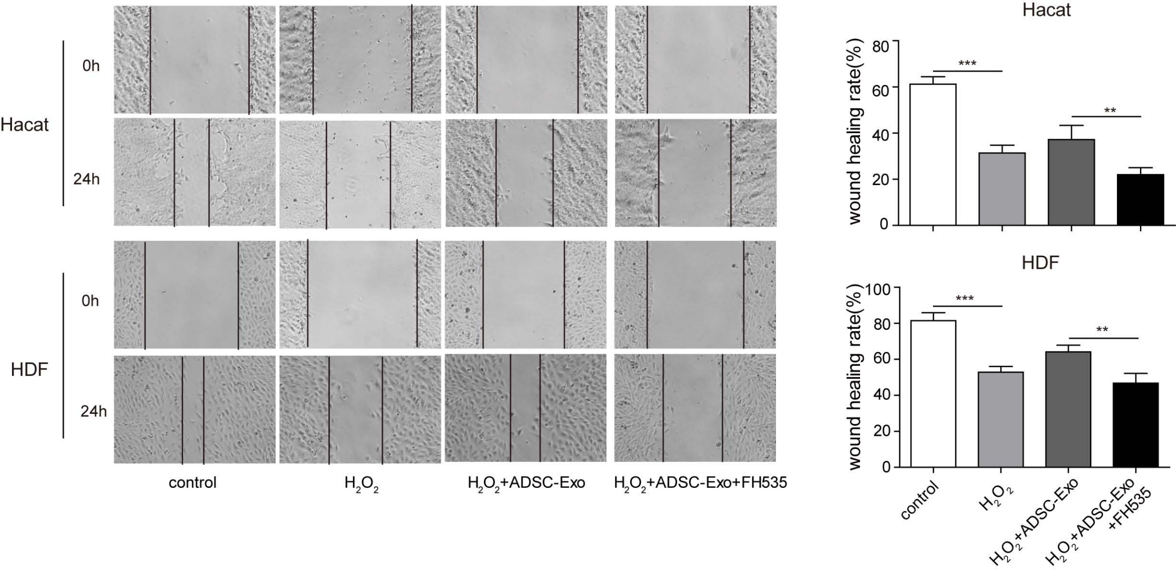

Hacat
0h
24h
HDF
0h
24h
control
H_2O_2
H_2O_2+ADSC-Exo
H_2O_2+ADSC-Exo+FH535
Hacat
wound healing rate(%)
0
20
40
60
80
***
**
HDF
wound healing rate(%)
0
20
40
60
80
100
***
**
control
H_2O_2
H_2O_2+ADSC-Exo
H_2O_2+ADSC-Exo
+FH535

Supplementary Figure S8 Migration of HaCaT and HDF cells treated with FH535 was performed using the scratch wound healing assay.
